# Letter from C. L. Norton, Dentist of New York, to the Baltimore Editor

**Published:** 1844-06

**Authors:** 


### ARTICLE VII.

### *Letter from* C. L. Norton, *Dentist of New York, to the Baltimore Editor.*

*New York, Dec. 20th* 1843.

Dear Sir:—I herewith send you a tooth extracted by me on the 11th inst. As you will observe, it is a good specimen of an injected tooth, caused by inflammatory action. This tooth when first extracted was of a tolerably bright red color, throughout nearly the whole extent of its bony structure; at the extreme point of the root, however, it had retained its natural healthy appearance. During the few days it has been in my possession, it has faded considerably, and probably in time will nearly or quite resume its natural color.

Instances of this kind so rarely occur, that I have thought it not amiss to give a brief history of this case, hoping it may not be altogether uninteresting. The facts, then, are as follows:

A young woman of about twenty years of age, a servant in my employ, requested me to examine a tooth, which she said was somewhat painful. This was about six weeks since. On examination I found the tooth considerably decayed, but the internal or nerve cavity not yet exposed; I, at this time, removed a portion of the decay, but the tooth being exceedingly sensitive, I could not prepare it fully to receive a filling. I applied to it a small portion of *arsenic*, and two days after, prepared and filled the cavity with *tin*. At the time of filling, the soreness had entirely disappeared, the tooth was firm in its socket, and to all appearance in a healthy and proper state to receive a plug. The operation of plugging was performed with great care, guarding against pressure in such a manner as to compress the *nerve* or injure the *periosteum*. The tooth thus filled continued healthy and useful for about four weeks. At the end of this time, however, it became painful and extremely sore to the touch, and the patient suffered intensely with it for some three days before I was informed of the fact. I then examined the tooth, found the periosteum and neighboring gum to be very highly inflamed, the latter excoriated, the tooth discolored and of a reddish aspect. From the appearance of the gum, I supposed the patient had endeavored to relieve herself from the pain by the application of some *tooth-ache remedy*, and charged her with it; she however stoutly denied the charge, but acknowledged that she had "picked it with a pin."

Convinced of the impracticability, if not of the impossibility of reducing the inflammation and thereby saving the tooth, and encouraged also by the fact that it could be very well spared, (it being a dens sapientiæ, and as the remaining teeth were in a sound and perfect state, she having previously lost but one, and that the corresponding one of the opposite side to this which I herewith enclose,) I extracted it, and found it to be as you see, a good specimen of an "injected tooth."

It may not be out of place here to remark that this is another added to the many incontestible proofs of the vascularity of the teeth, and of their being endowed with vessels of circulation, notwithstanding the opinions of the great Hunter, and other eminent anatomists. But as this is a matter so generally conceded by all well informed practitioners of the present day, I will not enlarge upon it, but content myself with propounding a few inquiries. And,

*First.* Did inflammation, in this instance, proceed from the pressure of the filling upon the thin plate of bone covering the internal pulp, and thereby compressing and irritating that substance? Or,

*Second.* Did the dose of *arsenic* produce the inflammation, by being taken up by the vessels, and conveyed to the nerve through the intervening plate of bone? Or,

*Third.* Was the inflammation induced by the external irritation of the gum and periosteum, by "picking at it with a pin," (or the application of some other substance,) and which might have irritated the gum as the appearance indicated; and if so, did the internal membrane sympathize? And,

*Fourth.* In intense inflammation of the *periosteum,* is it possible for the vessels of circulation of the tooth to become distended and surcharged with red blood to such a degree as to give to its whole bony structure the appearance of a perfectly injected substance, without the aid or sympathy of the vessels occupying the internal cavity; or must *both these vessels* and the *periosteum* be highly inflamed at the *same time,* to produce this effect?

### ARTICLE VIII.

*Tartar, or Calculus of the Mouth.* Translated from the French of Dellabarre, by * A *

